# Translation: Aesthetic Values of the Buccal Fat Pad Excision in Middle Aged Patients

**DOI:** 10.1093/asjof/ojac072

**Published:** 2022-09-15

The English version is available here.

